# Cerebrospinal fluid level of Nogo receptor 1 antagonist lateral olfactory tract usher substance (LOTUS) correlates inversely with the extent of neuroinflammation

**DOI:** 10.1186/s12974-018-1084-x

**Published:** 2018-02-17

**Authors:** Keita Takahashi, Hideyuki Takeuchi, Yuji Kurihara, Hiroshi Doi, Misako Kunii, Kenichi Tanaka, Haruko Nakamura, Ryoko Fukai, Atsuko Tomita-Katsumoto, Mikiko Tada, Yuichi Higashiyama, Hideto Joki, Shigeru Koyano, Kohtaro Takei, Fumiaki Tanaka

**Affiliations:** 1 Department of Neurology and Stroke Medicine, Yokohama City University Graduate School of Medicine, 3-9 Fukuura, Kanazawa-Ku, Yokohama, 236-0004 Japan; 2 Molecular Medical Bioscience Laboratory, Department of Medical Life Science, Yokohama City University Graduate School of Medical Life Science, Suehiro-cho 1-7-29, Tsurumi-Ku, Yokohama, 230-0045 Japan

**Keywords:** Lateral olfactory tract usher substance, Neuroinflammation, Biomarker, Nogo receptor, Multiple sclerosis, Meningitis

## Abstract

**Background:**

Although inflammation in the central nervous system is responsible for multiple neurological diseases, the lack of appropriate biomarkers makes it difficult to evaluate inflammatory activities in these diseases. Therefore, a new biomarker reflecting neuroinflammation is required for accurate diagnosis, appropriate therapy, and comprehension of pathogenesis of these neurological disorders. We previously reported that the cerebrospinal fluid (CSF) concentration of lateral olfactory tract usher substance (LOTUS), which promotes axonal growth as a Nogo receptor 1 antagonist, negatively correlates with disease activity in multiple sclerosis, suggesting that variation in LOTUS reflects the inflammatory activities and is a useful biomarker to evaluate the disease activity. To extend this observation, we analyzed the variation of LOTUS in the CSF of patients with bacterial and viral meningitis, which are the most common neuroinflammatory diseases.

**Methods:**

CSF samples were retrospectively obtained from patients with meningitis (*n* = 40), who were followed up by CSF study at least twice, and from healthy controls (*n* = 27). Patients were divided into bacterial (*n* = 14) and viral meningitis (*n* = 18) after exclusion of eight patients according to the criteria of this study. LOTUS concentrations, total protein levels, and CSF cell counts in the acute and recovery phases were analyzed chronologically. We also used lipopolysaccharide-injected mice as a model of neuroinflammation to evaluate LOTUS mRNA and protein expression in the brain.

**Results:**

Regardless of whether meningitis was viral or bacterial, LOTUS concentrations in the CSF of patients in acute phase were lower than those of healthy controls. As the patients recovered from meningitis, LOTUS levels in the CSF returned to the normal range. Lipopolysaccharide-injected mice also exhibited reduced LOTUS mRNA and protein expression in the brain.

**Conclusions:**

CSF levels of LOTUS correlated inversely with disease activity in both bacterial and viral meningitis, as well as in multiple sclerosis, because neuroinflammation downregulated LOTUS expression. Our data strongly suggest that variation of CSF LOTUS is associated with neuroinflammation and is useful as a biomarker for a broader range of neuroinflammatory diseases.

## Background

Inflammation in the central nervous system (CNS) is associated with a wide range of neurological diseases. In particular, infection and autoimmunity cause acute or chronic inflammation in the CNS [1]. However, it is difficult to evaluate neuroinflammation in these diseases, in large part because of the lack of biomarkers suitable for judging their activities [2]. Although total protein concentrations or cell counts in the cerebrospinal fluid (CSF) are the most commonly used biomarkers for neuroinflammation in CNS diseases, they do not always vary in parallel with disease activities. For example, multiple sclerosis (MS), one of the most common neuroinflammatory diseases caused by an autoimmune mechanism, exhibits poor variation in conventional CSF biomarkers [3], making precise evaluation of disease activity difficult [4]. To complement CSF biomarkers, imaging biomarkers have recently been developed, including magnetic resonance imaging with ultra-small superparamagnetic iron oxide, a novel contrast agent, and positron-emission tomography using [^11^C] *(R)*-PK11195 ligand. These new markers now play an important role in evaluation of the activities of neuroinflammatory diseases, including MS [3, 5]. However, the availability of cutting-edge imaging techniques is generally limited, even in the advanced treatment hospitals and especially in emergency situations. Therefore, the utility of currently available biomarkers for evaluating neuroinflammation is unsatisfactory, creating a demand for new and practical biomarkers.

Recently, we showed that lateral olfactory tract usher substance (LOTUS, also called Crtac1B) is critical for axonal growth. LOTUS acts as a Nogo receptor 1 (NgR1) antagonist, preventing Nogo from binding to NgR1 [6–8]. Furthermore, we showed that LOTUS in the human CSF closely associated with the disease activity of MS, a representative neuroinflammatory disease accompanied by neurodegeneration [9–11]. Because various molecules associated with axonal growth during brain development mediate not only axonal guidance but also immune responses [12–17], our previous findings suggest that LOTUS is intimately involved in neuroinflammation, as well as axonal growth and/or degeneration in MS [18]. Therefore, we hypothesized that variations of LOTUS concentrations in the CSF may also reflect disease activity in other neuroinflammatory diseases.

In this study, to investigate the relationship between LOTUS and neuroinflammation, we analyzed the variation of LOTUS concentrations in CSF of patients with bacterial and viral meningitis. These patients’ clinical courses were objectively verified using established CSF markers such as total protein concentration, cell counts, and glucose, in addition to clinical findings, and we also performed comparisons between the acute and recovery phases in the same patients. Furthermore, we investigated the mechanisms underlying the observations in human patients using lipopolysaccharide-injected mice as a disease model of neuroinflammation.

## Methods

### Participants

All patients enrolled in this study had underwent neurological evaluation at our university hospital and been diagnosed as meningitis. Between January 2008 and September 2017, CSF samples were obtained retrospectively from 40 patients who had been followed up for CSF study at least twice, after initial sampling in the acute phase, and 27 healthy controls. Patients with aseptic (*n* = 20), bacterial (*n* = 15), or tuberculous (*n* = 5) meningitis were classified as follows [19]. Patients with normal CSF glucose level (≥ 40% of plasma glucose level), monocyte-predominant CSF pleocytosis, or viral antigens were classified into the aseptic group (*n* = 20). Patients in the aseptic group with infectious sign (*n* = 18) were defined as viral meningitis, and two patients were ruled out due to lack of infectious sign. Patients with reduced CSF glucose level (< 40% of the plasma glucose level), neutrophil-predominant CSF pleocytosis, or positive microbiological culture were classified into the bacterial group (*n* = 14), and one patient was excluded due to complications of hydrocephalus. Patients with *Mycobacterium tuberculosis* gene-positive CSF, as confirmed by polymerase chain reaction, were classified into the tuberculous meningitis group (*n* = 5). In these patients, prolonged recovery period [20], relatively unchanged CSF total protein and cell counts over the short term made it difficult to judge disease activity. Therefore, we excluded patients with tuberculous meningitis from this study. No patients presented with Cryptococcus antigen or other fungal antigens in the CSF. All patients improved clinically during observation.

When the follow-up CSF was collected from the same patient twice or more, the last sample was analyzed between January 1, 2008, and January 1, 2014.

### Animals and drug treatment

We used 8- to 10-week-old male C57BL/6J mice obtained from Japan SLC (Hamamatsu, Japan). A neuroinflammation response was induced by intraperitoneal injection of 40 mg/kg lipopolysaccharide (LPS; L3129, Sigma-Aldrich, St. Louis, MO, USA); phosphate-buffered saline (PBS) was administered as a negative control. Mice were analyzed 24 or 48 h after treatment.

### RNA extraction and reverse transcription–polymerase chain reaction (RT-PCR)

Total RNA was extracted from the brains of LPS- or PBS-injected mice using the miRNeasy Mini Kit (Qiagen, Valencia, CA, USA) and reverse-transcribed using the SuperScript III First-Strand Synthesis SuperMix for RT-PCR (Life Technologies, Rockville, MD, USA). Expression levels of the genes encoding LOTUS (*Lotus*) and hypoxanthine phosphoribosyltransferase 1 (*Hprt1*) were evaluated by quantitative PCR (qPCR) using Power SYBR Green PCR Master Mix (Thermo Fisher Scientific, Wilmington, DE, USA) on a LightCycler® 96 SW 1.1. (Roche, Basel, Switzerland). Relative expression levels were determined using the ΔΔC_T_ method; the levels of the mRNAs of interest were normalized against the geometric mean level of *Hprt1* mRNA The following specific primer sets were used:

Mouse *Lotus* sense: 5′-CATGTTCACTGCAGTCACCAA-3′;

Mouse *Lotus* antisense: 5′-TTATTGGTGTTGAGAAAGTAGAT-3′;

Mouse *Hprt1* sense: 5′-CCTAAGATGAGCGCAAGTTGAA-3′;

Mouse *Hprt1* antisense: 5′-CCACAGGACTAGAACACCTGCTAA-3′.

### Assessments of LOTUS protein

LOTUS protein levels in patients’ CSF and mouse brains were analyzed by immunoblotting using a commercially available specific antibody (AF5234, R&D System, Minneapolis, MN, USA). The detailed methodology of this assay is described elsewhere [9]. Experiments using CSF were performed in triplicate. The mean value and coefficient of variation were calculated to certificate intra-assay reproducibility. Relative levels of LOTUS protein in mouse brains were quantified relative to β-actin, used as a loading control. The experiments were carried out in five independent trials.

### Statistical analysis

Statistical analysis was performed with Wilcoxon matched-pairs signed-rank test or Student’s *t* test as appropriate, using GraphPad Prism version 6.0 (GraphPad Software, La Jolla, CA, USA). *P* < 0.05 was considered statistically significant.

## Results

The characteristics of the patients enrolled in this study are summarized in Table 1. As shown in Table 1, the timing of sample collection did not differ significantly between viral and bacterial meningitis in either the acute or recovery phases (*p* = 0.31 and 0.53, respectively).

**Table 1:** Demographics and clinical characteristics of patients and controls

	Viral meningitis		Bacterial meningitis		Control
Number of patients	18		14		27
Age (year)	37.6 ± 14.9		64.1 ± 21.5		44.4 ± 20.5
Sex (male/female)	6/12		5/9		15/12
	Acute phase	Recovery phase	Acute phase	Recovery phase	–
Time of CSF sampling (days from onset)	3.4 ± 2.2	12.4 ± 4.8	2.5 ± 2.7	13.9 ± 7.9	–
CSF total protein (mg/dl)	85.3 ± 36.6	46.6 ± 21.4	298.5 ± 266.5	78.6 ± 55.8	30.8 ± 12.4
CSF cell count (μl)	185.9 ± 161.9	78.0 ± 90.0	1017.0 ± 1592.0	37.6 ± 45.0	2.4 ± 2.7
CSF LOTUS level (ng/ml)	133.5 ± 44.2	193.2 ± 83.5	114.2 ± 46.6	187.5 ± 92.1	192.1 ± 47.1

**Fig. 1:**
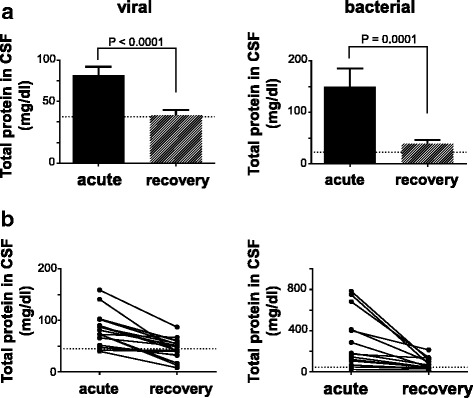
Comparison of total protein in CSF between acute and recovery phases. **a** Total protein in CSF of patients with viral (*n* = 18) and bacterial meningitis (*n* = 14). **b** Individual variation in CSF total protein. Horizontal dashed lines (45 mg/dl) represent the normal value. Values are means ± s.e.m. *P* values were calculated by Wilcoxon matched-pairs signed-rank test

**Fig. 2:**
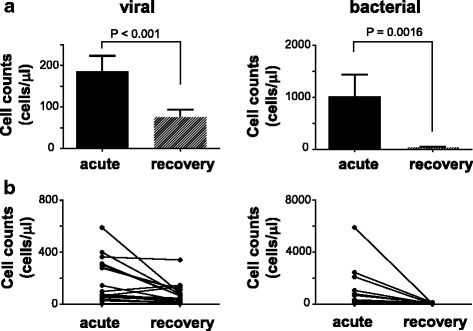
Comparison of cell counts in CSF between acute and recovery phases. **a** Cell counts in CSF of patients with viral (*n* = 18) and bacterial meningitis (*n* = 14). **b** Individual variation in CSF cell counts. Values are means ± s.e.m. *P* values were calculated by Wilcoxon matched-pairs signed-rank test

First, we examined conventional CSF biomarkers of meningitis, such as total protein and cell counts, in the acute and recovery phases in the same patients. Objective improvements in these markers and clinical recovery were confirmed in all patients with meningitis (Figs. 1 and 2).

**Fig. 3:**
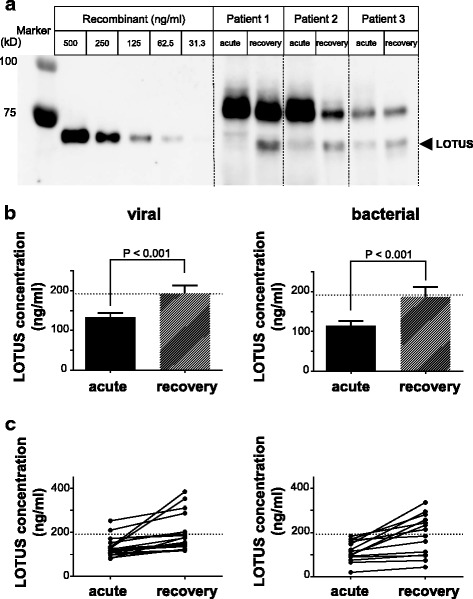
Comparison of LOTUS concentrations in CSF between acute and recovery phases. **a** Representative immunoblots of CSF LOTUS in acute and recovery phase of three patients with meningitis. The concentration of LOTUS in the CSF was calculated using a standard curve generated from blot band intensities of recombinant protein. **b** LOTUS concentration in CSF of patients with viral (*n* = 18) and bacterial meningitis (*n* = 14). Horizontal dashed lines (192.1 ng/ml) represent mean LOTUS concentration in healthy controls. Values are means ± s.e.m. **c** Individual variation in CSF LOTUS concentration. Horizontal dashed lines (192.1 ng/ml) represent mean LOTUS concentration in healthy controls. *P* values were calculated by Wilcoxon matched-pairs signed-rank test

We next assessed the CSF LOTUS concentration in the acute and recovery phases by immunoblot analysis (Fig. 3a). The standard value for CSF LOTUS concentration was defined as ≥ 192.1 ng/ml, based on the results in healthy controls (Table 1). Significant differences in LOTUS concentrations were observed between the acute and recovery phases regardless of whether the meningitis was viral or bacterial. The CSF levels of LOTUS significantly decreased in the acute phase of viral and bacterial meningitis, but returned to the normal level in the recovery phase (Fig. 3b). Individual analysis also revealed an increase in LOTUS concentration in all patients with both viral and bacterial meningitis (Fig. 3c).

**Fig. 4:**
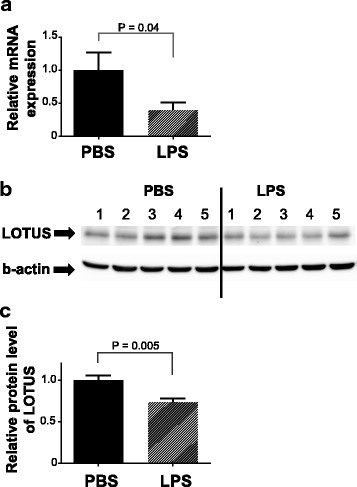
mRNA and protein levels of LOTUS in LPS-treated mouse brains. **a** qPCR data for relative expression of *Lotus* mRNA in LPS-treated mouse brains. Three samples were obtained 24 h after LPS injection. **b** Representative immunoblots of LOTUS. **c** Relative protein levels of LOTUS in brain of LPS-treated mice. Five samples were collected 48 h after LPS injection. Values are means ± s.e.m. *P* value was calculated by Student’s *t* test

Finally, we asked whether neuroinflammation downregulates *Lotus* mRNA expression in the brain, using LPS-injected mice as a model. *Lotus* mRNA expression was downregulated in LPS-inflamed brains, followed by a decrease in LOTUS protein levels (Fig. 4).

## Discussion

Myelin components in the CNS are important environmental factors that exert an inhibitory effect on axonal growth. Nogo, myelin-associated glycoprotein (MAG), and oligodendrocyte myelin glycoprotein (OMgp) are representative myelin components [21]. These molecules bind to a common receptor, NgR1, and prevent axonal growth, thereby leading to neuronal degeneration [22]. Because LOTUS endogenously functions as an NgR1 antagonist, preventing Nogo from binding to NgR1, it can promote axonal growth and is thus a promising therapeutic target for neural regeneration [6]. These molecules have attracted attention due to their relationship with MS pathogenesis and also as therapeutic targets. In particular, recent studies have demonstrated that the activation of NgR1-mediated signaling plays a substantial role in axonal degeneration in MS. Furthermore, a previous study by our group showed that LOTUS levels in the CSF correlated inversely with disease activity of MS, suggesting that its reduction during the active phase may be involved in the pathogenesis of MS. Interestingly, a recent study suggested that Nogo and NgR1, as well as other molecules, are related not only to axonal guidance but also to the immune system. This observation demonstrated that lymphocytes express NgR1 and that Nogo–NgR1 signaling can alter lymphocyte phenotypes [17]. Accordingly, we hypothesized that LOTUS is also involved in the immune system and thus represents a candidate novel biomarker for disease activity associated with neuroinflammation. In this study, we elucidated the correlation between the CSF LOTUS level and neuroinflammation in meningitis, one of the most common neuroinflammatory diseases. Our results indicated that, similar to Nogo, LOTUS is involved in the immune system as well as playing a role in axonal guidance.

Currently, total protein concentration and cell counts in the CSF are widely used as biomarkers to evaluate disease activity, although they are not necessarily appropriate for monitoring neuroinflammation in most CNS diseases. For evaluation of meningitis, these conventional markers are well established; therefore, we first confirmed improvement of meningitis in reference to these CSF biomarkers, in addition to the clinical neurological findings. Then, we chronologically compared the CSF LOTUS concentration between acute and recovery phases. We observed that the LOTUS level was reduced in acute phase, followed by recovery to the healthy control level, accompanied by clinical improvement. These data demonstrated that the CSF LOTUS concentration is inversely correlated with the disease activity of meningitis, as we previously reported in MS.

We then considered two possible mechanisms for a decrease in LOTUS: (1) downregulation of *Lotus* mRNA expression by neuroinflammation, and (2) enhanced degradation of LOTUS protein. Accordingly, we investigated whether *Lotus* mRNA levels decreased with the severity of neuroinflammation in LPS-injected mice, which is widely used as an animal model for neuroinflammation [23–25]. As expected, LPS-treated mice exhibited a significant downregulation of *Lotus* mRNA, along with a reduced level of LOTUS protein, in the brain. These results indicated that the mechanism underlying reduced CSF LOTUS concentration is downregulation of *Lotus* mRNA expression in response to neuroinflammation.

Previous proteomic studies reported LOTUS in CSF from normal subjects [26], and another group reported that LOTUS was not detectable in the CSF of patients with viral meningitis and CNS sarcoidosis [27]. Indeed, we confirmed a reduction in CSF concentration of LOTUS in acute phase of three patients with CNS sarcoidosis (61.6 ± 21.1 ng/ml). Thus, these findings support our results that reduced LOTUS concentration is associated with neuroinflammation in the acute phase of the diseases.

Interleukin-6 (IL-6) concentration in the CSF is often used as a biomarker of neuroinflammation in bacterial meningitis and progressive neuro-Behçet’s disease [28–31]. However, the sensitivity of IL-6 is too low to estimate disease activity in non-progressive neuro-Behçet’s disease or MS [32–34], indicating that IL-6 is of limited utility as a biomarker of neuroinflammation.

In contrast to IL-6, LOTUS may reflect disease activity of wider range of CNS diseases such as viral and bacterial meningitis, CNS sarcoidosis, and MS. Thus, the CSF LOTUS level is potentially of great diagnostic value for estimating neuroinflammation in a variety of CNS diseases, and we hypothesize that LOTUS might be critical to the pathogenesis of these diseases.

## Conclusion

CSF levels of LOTUS correlated inversely with disease activity both in bacterial and viral meningitis, as in multiple sclerosis. Therefore, variation of CSF LOTUS is associated with neuroinflammation and is useful as a biomarker, leading accurate diagnosis, appropriate therapy, and comprehension of pathogenesis for a broader range of neuroinflammatory diseases.

## Abbreviations

CNS: Central nervous system
IL-6: Interleukin-6
LOTUS: Lateral olfactory tract usher substance
LPS: Lipopolysaccharide
MAG: Myelin-associated glycoprotein
MS: Multiple sclerosis
NgR1: Nogo receptor 1
OMgp: Oligodendrocyte myelin glycoprotein
PBS: Phosphate-buffered saline
PCR: Polymerase chain reaction
RT: Reverse transcription
